# Role of PFKM lactylation in glycolysis regulation in endometrial cancer cells

**DOI:** 10.1016/j.gendis.2024.101400

**Published:** 2024-08-30

**Authors:** Bin Wang, Jian Ma, Di Yang

**Affiliations:** a Department of Gynecology, Liaoning Cancer Hospital and Institute, Cancer Hospital of China Medical University, Shenyang, Liaoning 110042, China; b Department of Obstetrics and Gynecology, Shengjing Hospital of China Medical University, Shenyang, Liaoning 110004, China; c Department of Obstetrics and Gynecology, The First Affiliated Hospital of Dalian Medical University, Dalian, Liaoning 116011, China

The incidence of endometrial cancer (EC) is increasing, particularly in high-risk cases with poor prognosis.^1^ Reprogramming glucose metabolism is the main feature of energy metabolism in EC cells, resulting in substantial lactate accumulation.^2^ Recent research indicates that the accumulation of lactic acid during the metabolic process can serve as a substrate.^3^ The high concentration of lactate accumulated by metabolites can promote the malignant progression of tumor cells by modifying key proteins through lactylation.^4^^,^^5^ Therefore, the role of lactylation in the occurrence and development of EC is worth exploring. We found lactylation-related genes (*PFKM*, *TXN*, *AKR1A1*, *MECP2*, *MDH1*, *GPI*, *GOT2*, and *COX4I1*) that play a role in the occurrence and development of EC and constructed a lactylation score model. The lactylation score has a strong correlation with the clinicopathological features, immune cell infiltration, and genetic instability of EC based on the data in The Cancer Genome Atlas database. Our research showed that PFKM (phosphofructokinase, muscle) underwent lactylation *in vitro*, which promoted colony formation, invasion of Ishikawa cells, and angiogenesis of HUVEC cells. Our findings provide new molecular prognostic markers and potential therapeutic targets for EC, thereby contributing to the identification of potentially effective drugs for EC.

We identified 26 lactylation-related pathways from the Gene Set Enrichment Analysis (GSEA) database, among which six GSEA results showed up-regulation in EC tissue (Fig. S1A). Then, we identified eight prognostic lactylation-related genes and plotted a forest map (Fig. S1B). In addition, we plotted a copy number variant dumbbell diagram of single gene mutation frequency for the eight lactylation-related genes. The frequency of acquired copy number changes in *AKR1A1* (Aldo-Keto reductase family 1 member A1), *GPI* (glucose-6-phosphate isomerase), and *MECP2* (methyl-CpG binding protein 2) genes was lower than that of loss in the population of endometrial cancer patients (Fig. S1C). We constructed a waterfall plot (Fig. S1D) of the single-gene mutation frequency for the eight lactylation-related genes. Kaplan–Meier analysis for the overall survival of lactylation-related genes revealed statistically significant differences in the survival curves, except for *COX4I1* (cytochrome C oxidase subunit 4I1) (Fig. S1E). We constructed a prognostic network graph (Fig. S1F), which showed that *PFKM*, as a risk factor, positively correlated with other *MECP2* and *MDH1* (malate dehydrogenase 1) risk factors and significantly negatively correlated with the three favorable factors.

Cluster analysis of lactylation-related genes was used to divide all uterine corpus endometrial carcinoma (UCEC) samples into two clusters (Fig. S2A). Survival analysis showed significant differences between the two groups, with cluster 2 having a lower survival rate than cluster 1 (Fig. S2B). We used single sample gene set enrichment analysis to determine the proportions of the 28 different immune cell types in the two EC clusters, which showed that cluster 1 had a higher overall immune cell type than cluster 2 (Fig. S2C). The clinicopathological features heatmap showed that *PFKM* and *MECP2* were highly expressed in the cluster 2 group (Fig. S2D). Using the GSVA algorithm, the results showed that the pathways in cluster 1 were mainly focused on the exogenous metabolism pathway (Fig. S2E).

We performed *t*-distributed stochastic neighbor embedding dimensionality reduction on the eight lactylation-related genes and extracted their main features (Fig. S3A). The lactylation score of cluster 2 was considerably higher than that of cluster 1 (Fig. S3B). The high-expression lactylation score group had a poor prognosis and survival (Fig. S3C). Additionally, tumor mutation burden (TMB) analysis revealed a negative correlation (Fig. S3D) between the two groups of patients. The results showed that the high-expression lactylation score group had a lower TMB. Additionally, lactylation score and TMB showed a significant negative correlation (Fig. S3E). Here, we further classified the lactylation score group according to the level of TMB, and the results showed that patients with higher lactylation scores and lower TMB had worse prognosis survival (*P* < 0.05) (Fig. S3F–H).

We predicted patient survival by constructing a column chart to statistically score the clinicopathological features and lactate levels, and the results showed that poor prognosis, advanced tumor age, stage, and grade, and lymph node metastases were closely related to a high lactylation score (Fig. S4A–C). We estimated the half-maximal inhibitory concentration (IC50) of the predictive model for the four chemotherapy drugs in the TCGA dataset. Doxorubicin and cyclopamine showed higher sensitivity in the low-lactylation score group, whereas cisplatin and paclitaxel showed higher sensitivity in the high-lactylation score group (Fig. S4D).

According to the ESTIMA-TE algorithm, the high-lactylation group exhibited lower matrix and immune scores than the low-lactylation group, indicating higher interstitial and immune cell infiltration in the low-lactylation group (Fig. S5A). Analysis of the correlation between lactylation scores and immune cell infiltration revealed a negative correlation with CD4^+^ and CD8^+^ T-cell infiltration (Fig. S5B, C). We also studied the lactylation score and microsatellite instability, with the MicroSatellite Instability-High and MicroSatellite Stability groups having the lowest and highest lactylation scores, respectively (Fig. S5D). From the TIDE database, high levels of ips_ctla4_pos_pd1_pos indicate a response to both anti-CTLA-4 (cytotoxic T-lymphocyte associated protein 4) and anti-PD-1 (programmed death-1) therapy. We integrated the four immunophenotypic scores with the lactylation score group, and as shown by Fig. S5E, patients with low lactylation scores may be more sensitive to anti-CTLA-4 and anti-PD-1 antibody therapy (*P* < 0.05).

High expression of *PFKM*, *MECP2*, *MDH1*, and *GPI* in EC tissue was associated with poor prognosis (Fig. S1E). We conducted quantitative reverse transcription PCR to determine the expression of *PFKM*, *MECP2*, *MDH1*, and *GPI* in 19 normal endometrial tissues and 36 EC tissues (Fig. 1A). The results revealed significant overexpression of *PFKM*, *MECP2*, and *GPI* in EC tissues. To further investigate lactylation modification in PFKM, we performed liquid chromatography/mass spectrometry to identify the lactylated tyrosine K-678 site (Fig. 1B). PyMOL was used to visualize the PFKM and ATP complexes before and after lactate modification and these sites and the lactylation-modified sites on PFKM in the spatial structure (Fig. 1C). The PFKM levels were assessed in 20 normal endometrial and 30 EC tissues using immunohistochemistry. Moreover, the positive expression rate of PFKM expression was significantly higher in the EC group (70.0% [21/30]) than in the normal control group (40.0% [8/20]; *P* < 0.05; Fig. 1D and Table S2). High PFKM expression levels may be positively correlated with poor patient prognosis (Fig. 1E). We investigated the carcinogenic effects of PFKM lactylation in EC. Wild-type and mutant PFKM (K678R) plasmids were constructed for transfecting Ishikawa cells. Differences in glucose uptake and lactylation production between the wild-type and mutant PFKM (K678R) plasmids were determined using the *in vitro* glucose oxidase-peroxidase and lactate-oxidase methods, respectively. The results showed that after mutant PFKM (K678R) treatment, glucose uptake and lactate production in the cell line decreased (Fig. 1F, G). Moreover, western blot results showed reduced pan-Kla expression in the PFKM (K678R) group (Fig. 1H). Transfection of Ishikawa cells with mutant PFKM (K678R) plasmids significantly reduced proliferation, clonogenesis, and invasiveness of the EC cell line and reduced angiogenesis of HUVEC cells (Fig. 1I–L). Finally, we explored potential therapeutic drugs targeting PFKM using virtual screening. After docking, the data were scored based on energy (binding energy < −6 kcal/mol), and 1260 high-ranking compounds were selected (Table S3). By comparing the docking score, we identified six compounds making potential PFKM inhibitors (Fig. 1M, N).

**Figure 1:**
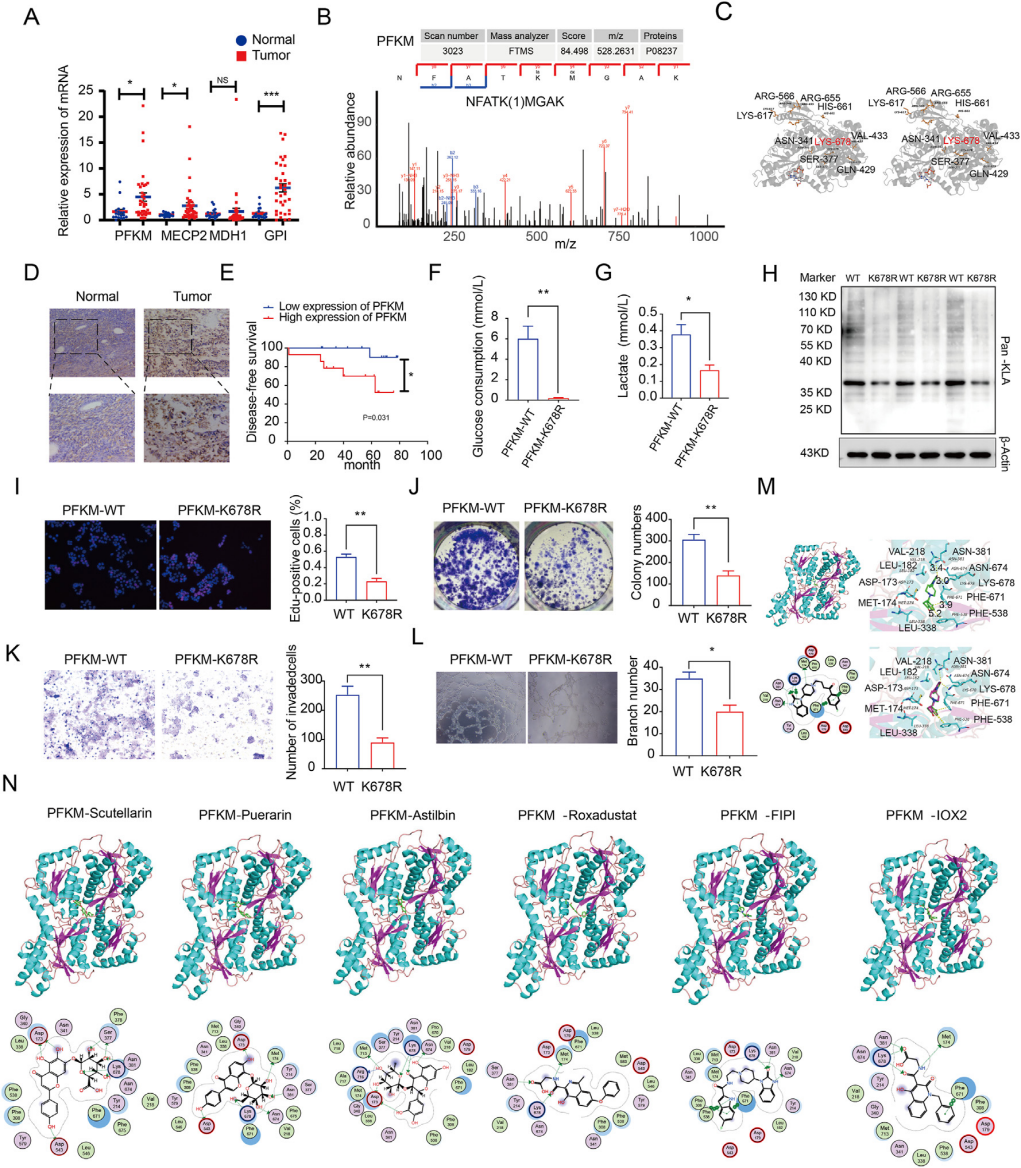
PFKM lactylation promotes the progression of endometrial cancer cells. **(A)** Expression of the four genes in 19 EC and 36 normal tissues was determined by quantitative reverse transcription PCR. **(B)** Collision-induced dissociation (CID) analysis for identifying modification sites. **(C)** Ribbon diagram of the human PFKM protein crystal structure. **(D)** PFKM expression in 30 EC and 20 normal tissues was determined with immunohistochemistry. **(E)** DFS curves for PFKM expression levels in 30 patients with EC. ∗*P* < 0.05. **(F)** Glucose uptake by Ishikawa cells. **(G)** Lactate production in Ishikawa cells. **(H)** Western blot analysis of pan-Kla abundance in response to PFKM treatment of Ishikawa cells. **(I, J)** Effect of PFKM expression on Ishikawa cell proliferation detected with EdU and colony formation assays. **(K)** Transwell assay was used to determine the number of invading cells. **(L)** Angiogenesis evaluation of HUVEC cells with a tube formation assay. **(M)** (a) Overall three-dimensional structure of the PFKM complex. The backbone of the protein was rendered in a tube and colored bright blue and pink. The ref-ligand was rendered as a stick and colored by elements. (b) Close view of the active site binding with the ref-ligand. Key residues interacting with the ref-ligand are rendered as sticks and colored in cyan. (c) The two-dimensional protein-ligand interaction diagram of the ligand-PFKM complex. Protein residues are rendered in a circle and colored based on their properties: green, hydrophobic residue; purple, polar residue. (d) Re-docking results of the ref-ligand with the PFKM target. **(N)** Binding modes of six ligands to the PFKM protein. Data are presented as mean ± standard error of the mean (*n* = 3 per group). ∗*P* < 0.05, ∗∗*P* < 0.01, and ∗∗∗*P* < 0.001 compared with the NC group.

In this study, our results suggest that lactylation-related pathways and lactylation of specific genes play significant roles in EC progression. Our findings provide potential therapeutic targets for EC.

## Ethics declaration

The study protocol was reviewed and approved by the Scientific Research and New Technology Ethical Committee of the Shengjing Hospital of China Medical University (ethical number: human: 2018PS251K). All authors consent to publish this paper and participate in this study.

## Author contributions

J.M. performed most of the experiments and contributed to manuscript writing. J.M. conceived the study, participated in its design and coordination, and helped draft the manuscript. B.W. participated in chart drawing and manuscript writing and revisions. J.M. and D.Y. performed the quantitative reverse transcription PCR and cell culture experiments. All authors read and approved the final manuscript.

## Conflict of interests

The authors declared no competing interests.

## Funding

This work was supported by the 10.13039/501100001809National Natural Science Foundation of China (No. 82203469) and the Liaoning Province Joint Fund General Project (China) (No. 2023-MSLH-166).

## Data availability

The datasets and code used during the current study are available from the corresponding author upon reasonable request.

## Figures and Tables

**figs1:**
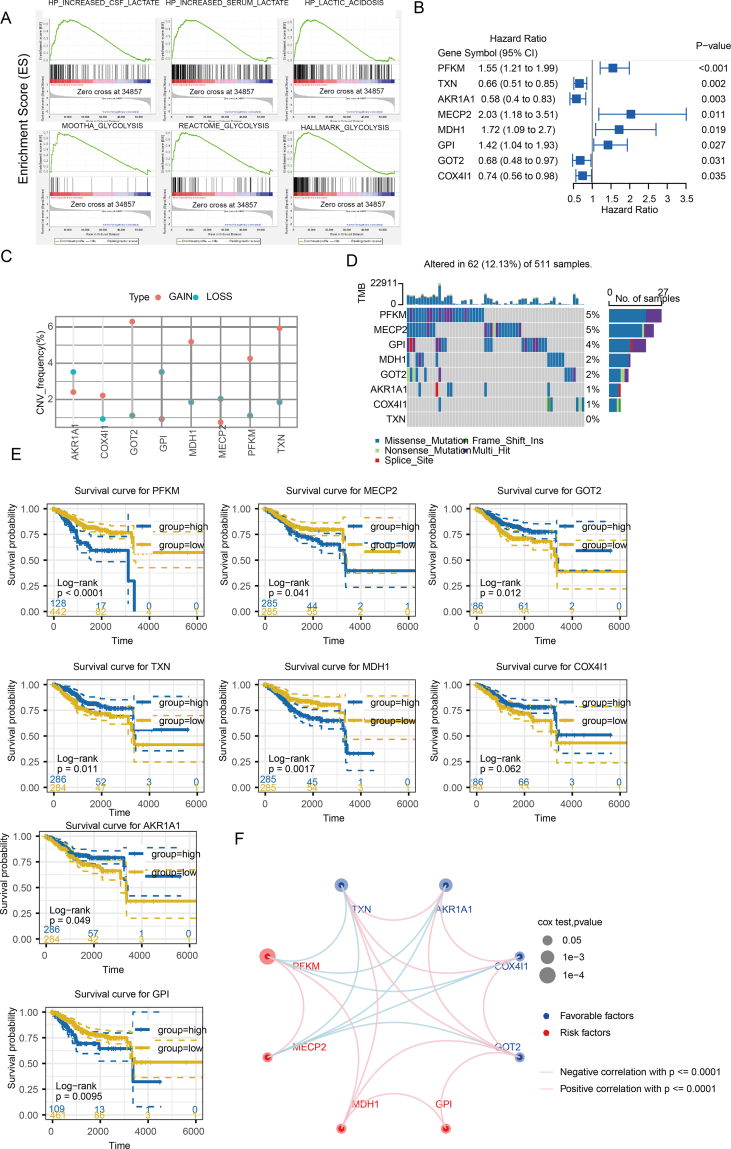


**figs2:**
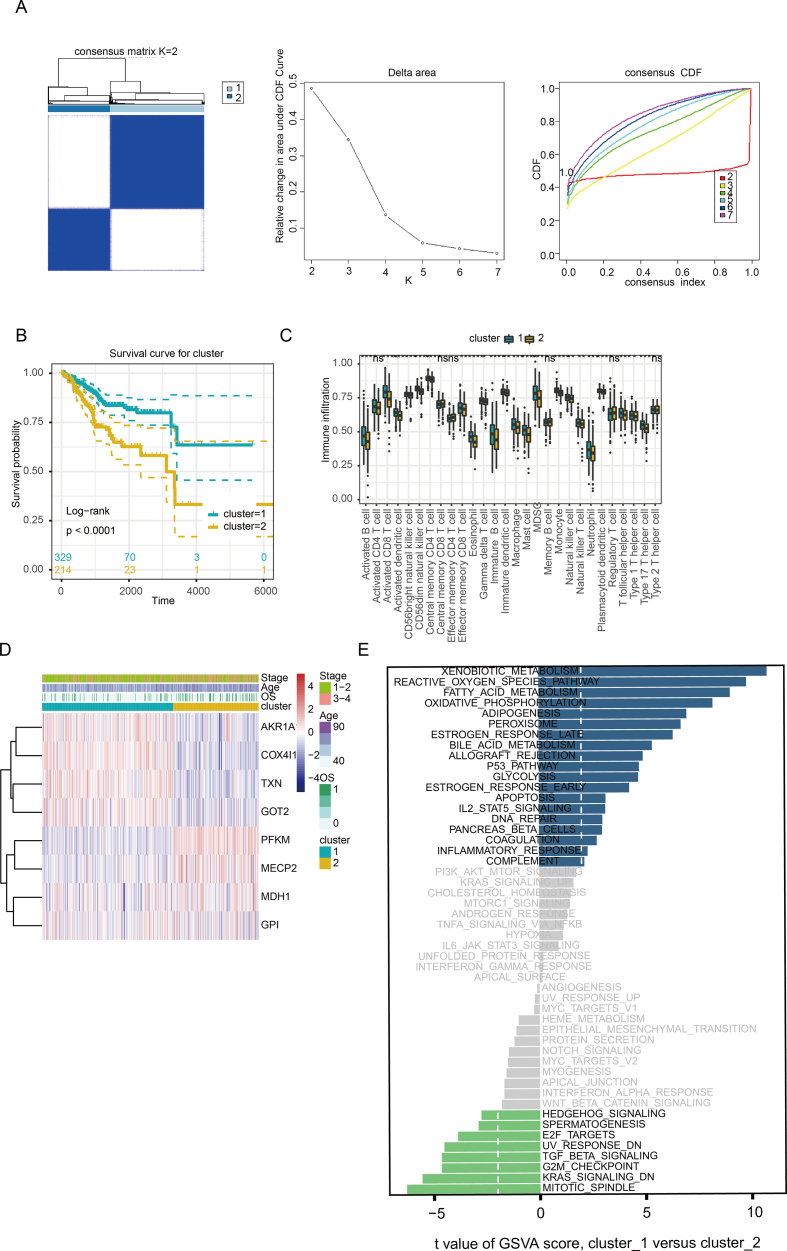


**figs3:**
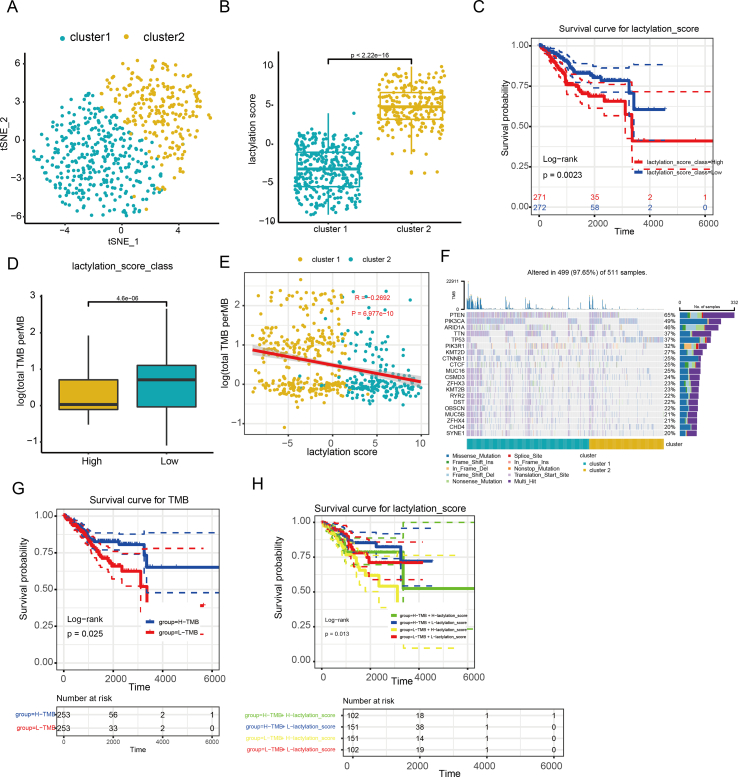


**figs4:**
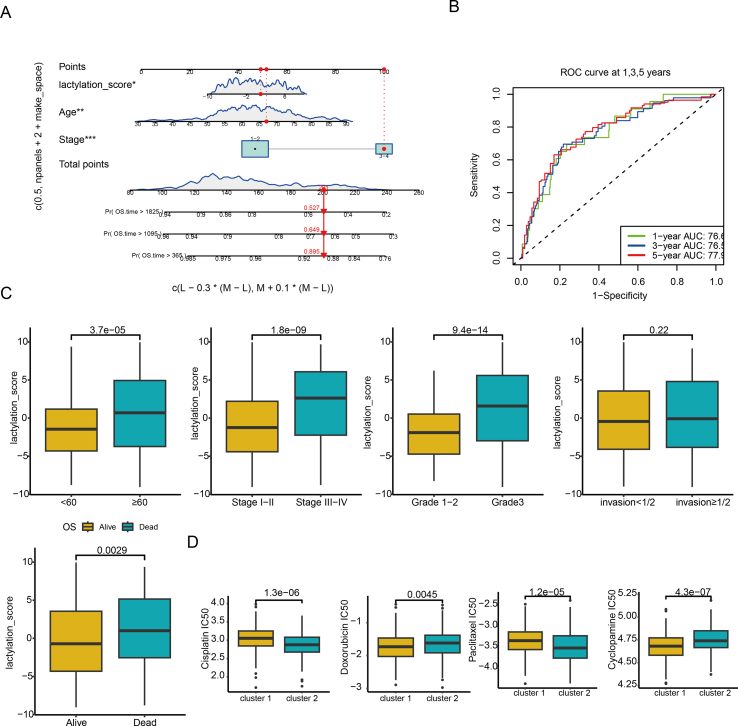


**figs5:**
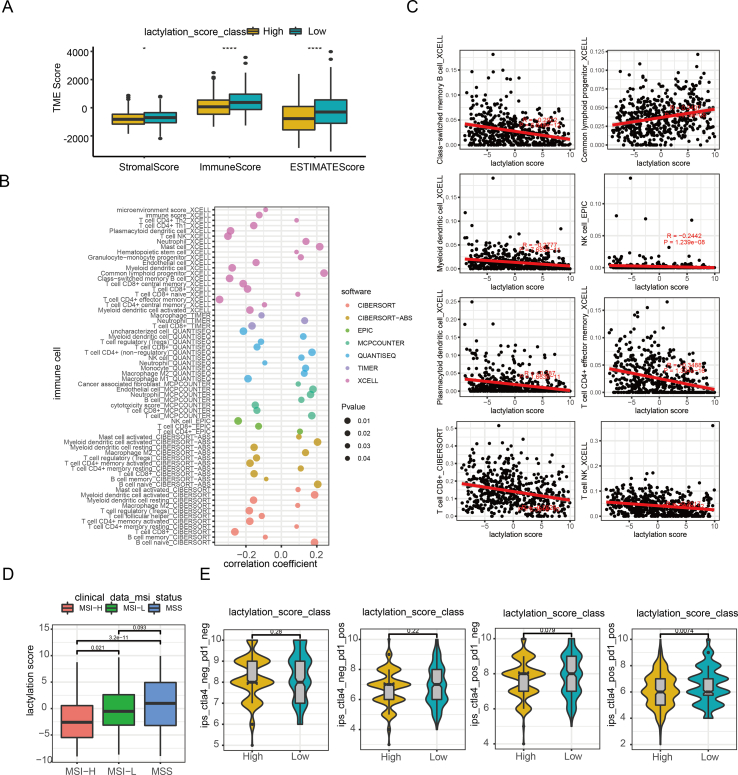


## References

[1] Crosbie E.J., Kitson S.J., McAlpine J.N., Mukhopadhyay A., Powell M.E., Singh N. (2022). Endometrial cancer. Lancet.

[2] Ippolito L., Morandi A., Giannoni E., Chiarugi P. (2019). Lactate: a metabolic driver in the tumour landscape. Trends Biochem Sci.

[3] Certo M., Tsai C.H., Pucino V., Ho P.C., Mauro C. (2021). Lactate modulation of immune responses in inflammatory versus tumour microenvironments. Nat Rev Immunol.

[4] Pandkar M.R., Sinha S., Samaiya A., Shukla S. (2023). Oncometabolite lactate enhances breast cancer progression by orchestrating histone lactylation-dependent c-Myc expression. Transl Oncol.

[5] Jiang J., Huang D., Jiang Y. (2021). Lactate modulates cellular metabolism through histone lactylation-mediated gene expression in non-small cell lung cancer. Front Oncol.

