# Supplementary material for: Role of PFKM lactylation in glycolysis regulation in endometrial cancer cells
Source: Genes Dis. 2024 Aug 30;12(3):101400. doi: 10.1016/j.gendis.2024.101400 (PMC11786832; doi:10.1016/j.gendis.2024.101400)
Supplement: Multimedia component 1 [file mmc1.docx]

**Supplementary Data**

**MATERIALS AND METHODS**

**Data retrieval and processing**

Using keywords (such as lactic acid, lactate, and glycolysis) to search the GSEA pathway data (December 2021; https://www.gsea-msigdb.org), the following pathways were obtained: lactic acid (lactate); lactation modification: glycolysis pathway information can be found in the GSEA lactation related pathway folder (merged, where gct represents the merged data). TCGA RNA seq expression profile, SNV mutation data, and CNV copy multiple variation data can be accessed from https://portal.gdc.cancer.gov/ as of December 2021. TCGA clinical information and MSI data can be downloaded from cBioportal (www.cbioportal. org/).

**Screening of lactylation-related genes for prognosis of EC**

By analysing differentially expressed genes in UCEC tumours and normal tissues using the Limma R package analysis and sorting the gene expression profile according to fold-change, GSEA was performed on 26 lactylation-related pathways, and six pathways were found to be tumour-enriched. Please refer to Gsea.1680457226131/gsea_Report_For_Tumor_ 1680457226131.tsv (qvalue<=0.05) for a detailed analysis. We conducted differential expression analysis and univariate Cox analysis using limmaR packages on the genes in these six pathways and ultimately identified eight prognosticlactylation-related genes. We calculated the copy number variation frequency of these genes based on changes in gene copy numbers in the UCEC samples from the TCGA database and visualized it using a lollipop chart. The maftoolsR package was used to draw waterfall charts for these genes. The KM survival curves of these genes were drawn using the surveyorR package. We constructed a column chart using the regplotR package for age, lactylation score, survival status, and tumour grade in the clinical data of UCEC and plotted ROC curves using the time ROCR package.

**Cluster analysis**

The UCEC tumour samples were clustered using the ConsensusClusterPlus R package, and k = 2 was established as the optimal grouping result (see ConsensusCluster for the results). A heat map was drawn using the pheatmapR package, and eight prognosticlactylation-related genes were visualised based on clinical data. ssGSEA was performed for 28 immune-related pathways (mmc3.sv) using the GSVA R package, and the results were visualised in a box plot using ggplot2. Differential expression pathways between the two groups of samples were analysed with the GSVA, GSVAbase, and Limma R packages, using the reference gene set (h. all. v2023.1. Hs. symbols. gmt).

**Developing a lactylation scoring model**

We quantified individual patients’ lactylation modification levels using tSNE. Feature selection was conducted on eight prognosticlactylation-related genes, and principal components 1 and 2 were extracted as the two main feature scores using the tSNER package. We calculated the lactylation score for each patient by summing the values of tSNE1i and tSNE2i [=∑ (tSNE1i+tSNE2i)]. The ssGSEA algorithm was used to analyse the correlation between the lactylation score and immune cells present in tissues. We analysed the relationship between cluster classification, lactylation score, and TMB. A combined survival analysis was performed using the survivalR and survivinerR packages for the high tumour mutation load, low tumour mutation load, high emulsification score, and low emulsification score groups. All patients with UCEC were divided into MSS and MSI groups using a cutoff of 0.4, and the lactylation score for each group was calculated. We downloaded the TCGA-UCEC Exclusion and Dysfunction scores from the TIDE database (<http://tide.dfci.harvard.edu/>) to analyse immune evasion and dysfunction in the high- and low- lactylation scoring groups.

**Chemical response prediction**

We used the FPKM RNA-seq expression profile of TCGA to predict chemotherapy efficacy in each group according to the pharmacogenomics database (Genomics of Drug Sensitivity in Cancer (GDSC), <https://www.cancerrxgene.org/>). Prediction was carried out using the R software package ‘pprophetic’, where the half-maximum inhibitory concentration (IC_50_) value of the sample was estimated using ridge regression, and the accuracy of the prediction was evaluated using 10-fold cross-validation, according to the GDSC training set.

**Comparison of immune cell subgroups among molecular subtypes of EC**

The markers of immune cells are derived from the paper: https://doi.org/10.1016/j.celrep.2016.12.019. Then, we estimate the relative enrichment of above gene set in two distinct clusters via ssGSEA.

**Sensitivity analysis of lactylation-score groups to immunotherapy**

Downloaded the immunophenotypic score of the UCEC cohort from the TIDE database, which assesses patient response to immune checkpoint inhibitor therapy and reflects the likelihood of immune escape in patients with tumor.

**LC‒MS/MS analysis and database search**

LC‒MS/MS analysis was performed by Jingjie PTM BioLabs (Hangzhou, China).

**RNA extraction and qRT-PCR**

TRIzol reagent (Takara, Shiga, Japan) was used to extract total RNA from cultured

cells and tissues. For real-time PCR analysis, the total RNA was reverse transcribed into cDNA using the PrimeScriptTM RT-PCR Kit (Takara) and SYBR® TB GreenTM

Premix Ex Taq II (Takara). Specific PCR primers were designed by Sangon Biotech

Co., Ltd. (Shanghai, China). The fold-change in expression was determined using the ∆∆Ct method, with GAPDH as an internal control. Primer sequences are listed in Supplementary Table S1.

**Immunohistochemistry**

Immunohistochemistry was used to detect PFKM (Proteintech, China). The scoring method for positive expression was based on whether the nucleus had a brownish-yellow or brown colour. Samples were scored as follows: no staining, 0; light yellow, 1; yellow, 2; brown or sepia, 3. The scores were assigned according to the percentage of positive cells, with a negative count denoted as 0. The percentage of positive cells was scored as follows: < 10%, 1; ≥ 10–50%, 2; > 50–75%, 3; ≥ 75 %, 4. The product of the two scores was taken as the total score, and the results were interpreted as follows: ≤ 2, negative; 3–4, weak positive (+); 5–8, medium positive (++); 9–12, strong positive (+++). Low expression was indicated by -/+, and high expression was indicated by ++/+++. The results were evaluated by two senior pathologists who were blinded to the patient data. Each sample was independently observed to determine the positive cell count and evaluate the background. In cases of disagreement, a third pathologist was consulted to provide a final decision.

**Cell culture**

Ishikawa cells were cultured in RPMI 1640 medium (Gibco, Carlsbad, CA, USA). Cell lines were obtained from the Institute of Biochemistry and Cell Biology, Chinese Academy of Sciences (Shanghai, China).

**Transfection of Ishikawa cells**

Construction of PFKM wild-type (WT) and PFKM mutant (K678R) plasmids (Syngen tech, Beijing, China) using Lipofectamine™ 3000 (Thermo Fischer Scientific, Carlsbad, CA, USA) as per the manufacturer’s instructions.

**Cell proliferation assay**

Ishikawa cells at the logarithmic growth phase were uniformly suspended and transferred to 96-well plates. A blank control with three parallel wells was included in each group. An EdU cell proliferation detection kit (RiboBio, Guangzhou, China) was used to assess the cell proliferation capacity as per the manufacturer’s instructions. Briefly, 50 μM of EdU mixed reagent was prepared, and the cells were incubated with it for 2 h. After the cells were fixed, DNA staining was performed, and cells were washed with PBS. Image acquisition and analysis were performed using a fluorescence microscope (Nikon, Japan) at 20× the original magnification.

**Cell invasion assay**

After sterilising the Transwell chamber, Matrigel (pore size 8 μm; Corning, NY, USA) was added and incubated at 37 °C overnight for the gel to solidify. Cells in the logarithmic growth phase were harvested, centrifuged, and the supernatant was discarded. Cells were resuspended in a serum-free medium. Subsequently, 800 μL of culture medium (containing 10% foetal bovine serum) was added to another 24-well plate, and the Transwell chamber was transferred into the 24-well plate. The cell suspension (5 × 10^4^ cells) was added to the upper chamber and incubated for 24 h at 5% CO_2_ and 37 °C. The Transwell chamber was then removed and the non-adherent cells in each well were removed, while the liquid in the upper chamber was discarded. After air-drying the wells, they were fixed with 4% paraformaldehyde. Crystal violet dye solution was added, and cells were rinsed with phosphate-buffered saline (PBS). Images were obtained using a microscope, and the cells were counted.

**Clonogenic assay for cell proliferation**

Ishikawa cells in each group were seeded at 500 cells/well in a 6-well plate. After 2 weeks of continuous culture, the culture medium was removed, the cells were washed with PBS, fixed with 4% paraformaldehyde for 20 min, stained with 0.2% crystal violet for 5 min, washed with pure water, and dried. The cells were observed under a microscope, and images were obtained. The clone formation rate was calculated using the following formula: (number of cell clones/total number of cells) × 100%.

**Tubule Formation Assay**

Fifty microliters of matrix glue was added to a 24-well plate, which was then incubated for 30 min. After transfection for 24 h, HUVEC were collected and resuspended. Then HUVEC cells were added to the pre-coagulated matrix glue for further culture in the incubator. After 12 h, the culture plate was photographed under an inverted microscope and Image J (National Institutes of Health, Germany) was used to calculate the length of the tubule.

**Protein molecular docking**

We chose the PFKM protein crystal structure (PDBID: 4OMT) with a resolution of 6.00 Å. lactylation modification was performed at the tyrosine K-678 site. Next, using AutoDock software, we checked for any missing atoms or residues in the protein crystal structure and rectified this by adding hydrogen atoms to the protein structure and assigning partial charges to each atom. We obtained the 3D structure of ATP from the PubCHEM database and added the hydrogen, charge, and roots to the PMV before saving the file. Using the Docking module, ATP was docked to the active sites of PFKM and K-678 lactylation -modified PFKM proteins. The complexes of PFKM and ATP before and after lactylation modification were visualised using PyMOL, and amino acids Arg429, Val433, Ser377, Lys678, Asn341, Arg566, Arg655, His661, Lys557, Lys617, and Asp591 were displayed in a rod-shaped pattern.

**Virtual screening of PFKM targets**

We used the PFKM structure (PDB ID: 4OMT; https://www.rcsb.org/) for molecular docking simulation. The protein structure was processed on the Maestro 11.9 platform, where 4951 compounds were protonated and the energy was minimised using the LigPrep module and the OPLS3e force field. Virtual filtering and optimisation were completed using the Glide module in the Schrödinger Maestro software. Protein processing was performed using the Protein Preparation Wizard module. We designated the K678 of the protein as the centroid of the 15 Å box. First, the selected ligands were docked to confirm the feasibility of the docking method. The crystal structure of the PFKM protein displayed high accuracy with no missing key residues. Initially, we selected K678 as the centroid for a 15Å box, and then established screening parameters. Due to the absence of suitable positive compounds as references (currently, no effective active compound targeting the PFKM protein have been identified), we conducted a preliminary rapid high-throughput screening of the database. We identified the compound Droperidol, which exhibited excellent protein-binding modes and docking scores. Consequently, we performed redocking of this compound to revalidate our docking parameter settings and to exclude the possibility of random errors. The results showed that the redocked conformation closely overlapped with the ligand from the initial screening (RMSD < 0.8 Å), thus we consider this screening method to be both rational and effective. To establish an appropriate docking approach for screening potential active compounds, we redocked the droperidol ligand to the PFKM-binding site. The dataset was then screened through SP docking to demonstrate the suitability of the SP docking template for the large-scale screening of compounds. Finally, the XP docking template was used to screen pre-ligands with higher scores determined by the SP method, which aimed to provide a better correlation between pose and scoring. The larger the absolute value of the docking score, the better the binding between the compound molecule and the crystalline protein.

**Statistical analysis**

All data processing, analyses, and plotting were performed using R v.4.2.1. The data between the two groups were compared using Student’s t-test, and the correlation coefficient was calculated using the Spearman method. The log-rank test was used to compare differences in survival curves between two or more groups. The estimated IC50 values of the EC stem cell subtypes were compared using the Kruskal–Wallis test. Kaplan–Meier curves were used to evaluate the survival time of patients with EC. GraphPad Prism 8 software (GraphPad Inc., La Jolla, CA, USA) was used for all statistical analyses. *P*<0.05 was considered statistically significant.

**Supplementary Table Information**

Supplementary Table S1. Primer sequences for qRT-PCR and primary antibodies used for the detection of protein expression.

Supplementary Table S2. The expression of PFKM in normal endometrial tissue and endometrial carcinoma tissue

Supplementary Table S3. Based on the energy score of the data, 1260 compounds with stable binding to PFKM target proteins were selected from the top rankings.

**Supplementary Figures Information**


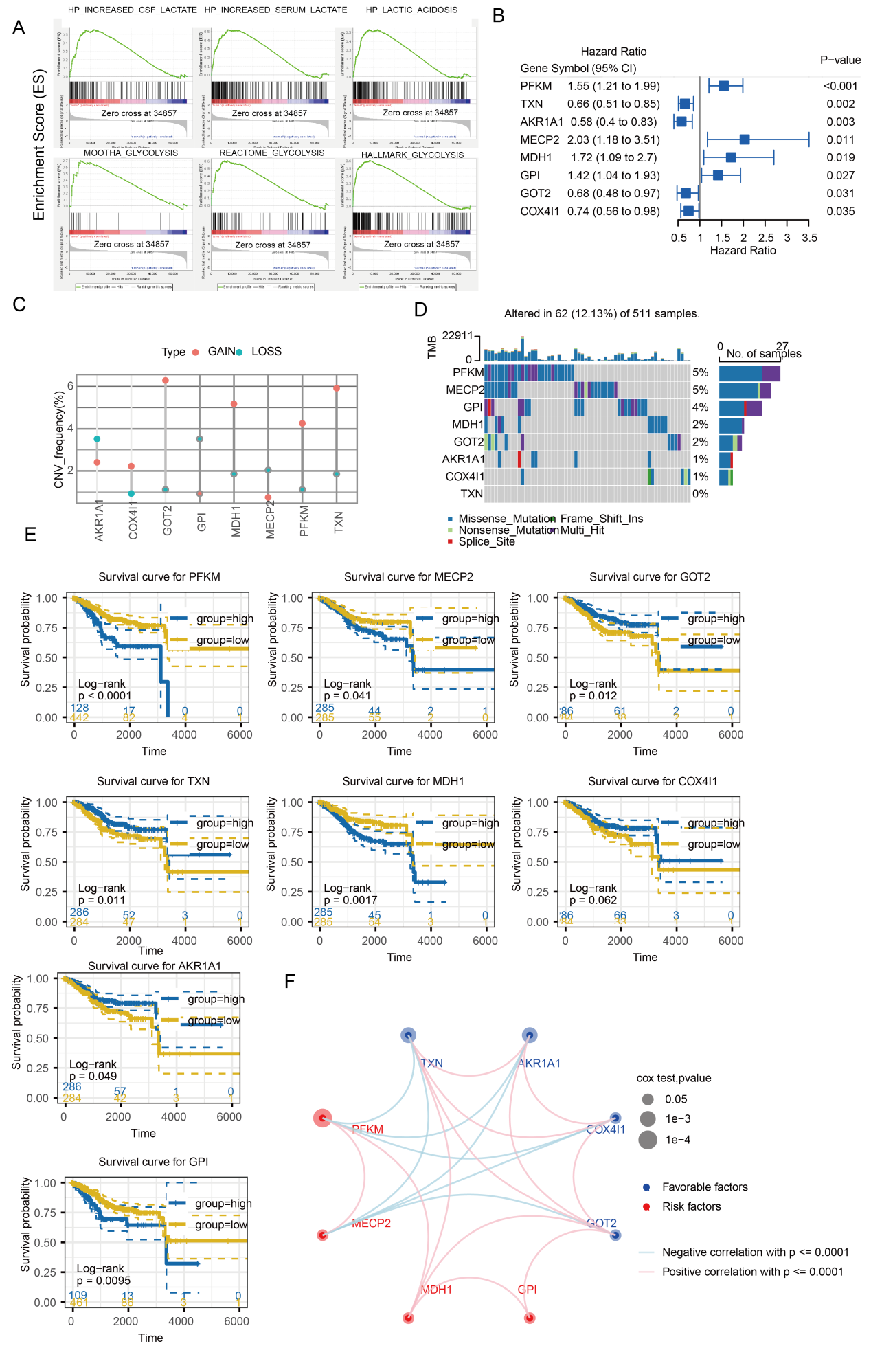


**Figure S1 Expression of lactylation-related genes in EC**

(A) Highly expressed GSEA pathways in EC tissues. (B) Univariate Cox regression analysis of lactylation-related genes. (C) The frequency of acquired copy number changes in this gene in the population is higher or lower than that of loss. The horizontal axis of the CNV dumbbell chart represents the frequency of copy number variation. (D) Waterfall plot of the mutation frequencies of lactylation-related genes. (E) Kaplan–Meier survival curves for the overall survival of lactylation-related genes in the TCGA cohort. The solid line represents the estimated survival probability at each time point, while the two dashed lines represent the upper and lower bounds of the estimated 95% confidence interval. (F) Prognostic network of lactylation-related genes.


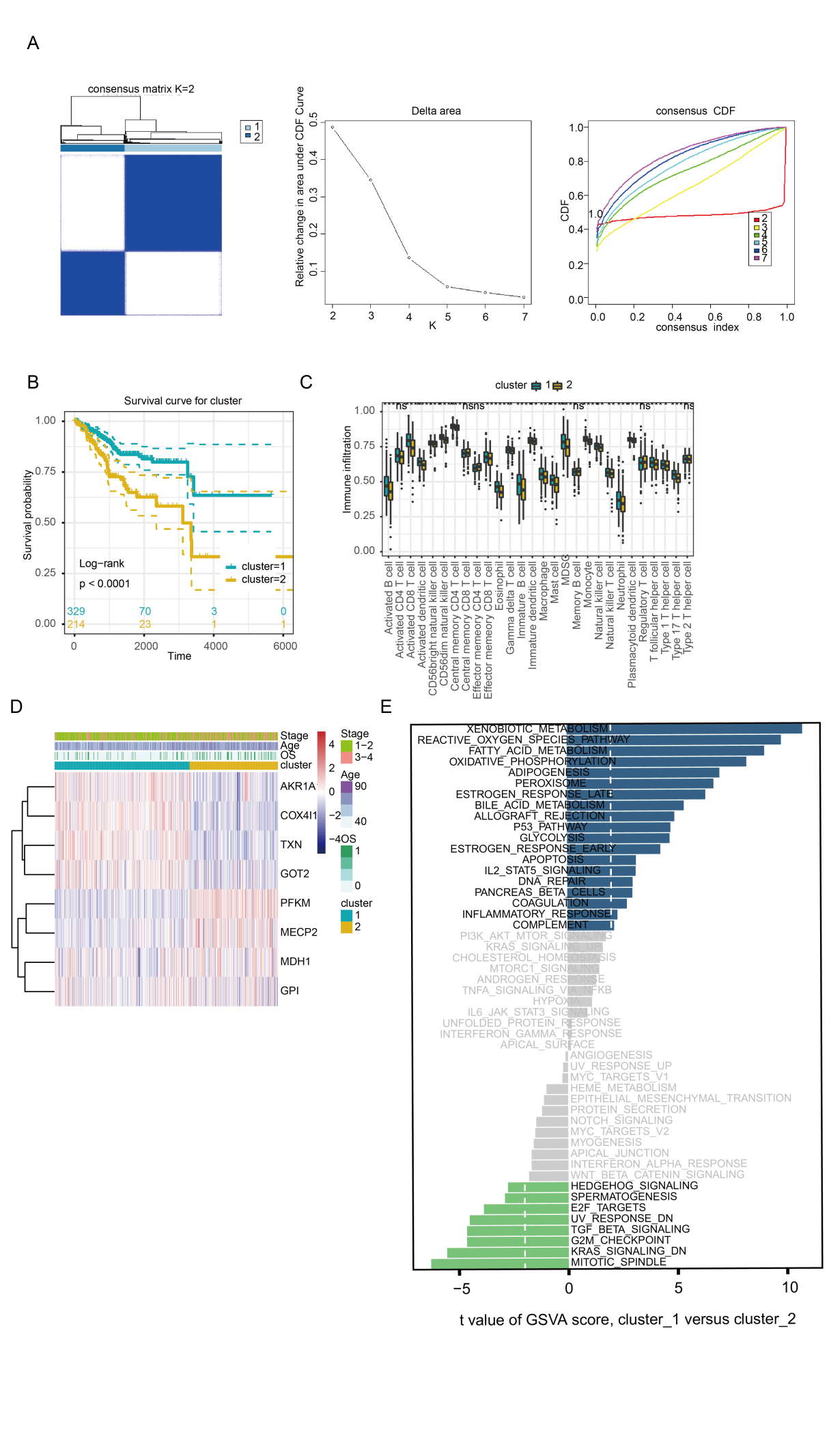


**Figure S2 EC classification of lactylation-related genes**

(A) Consistent clustering of the EC-TCGA cohort. When k = 2, the consensus score matrix of the EC samples is The consensus score between the two samples indicates that they are more likely to be assigned to the same cluster in different iterations. Area under CDF curve for k=2–7. (B) Kaplan–Meier survival analysis of the overall survival curves of the two clusters. (C) Analysis of immune cell infiltration in the two clusters. (D) The heatmap of normalized expression level of 8 lactylation related genes in EC. The red color represents the higher expression level of this gene. The top four colored bars in the figure are abbreviations of different groups of samples. Stage: AJCC Stage Groupings, stage 1-4 represent stage I-IV; OS: overall survival. (E) Using the GSVA algorithm, with h. all. v2023.1. Hs. symbols. gmt as the reference gene set, we compared the differentially expressed pathways in two UCEC clusters.


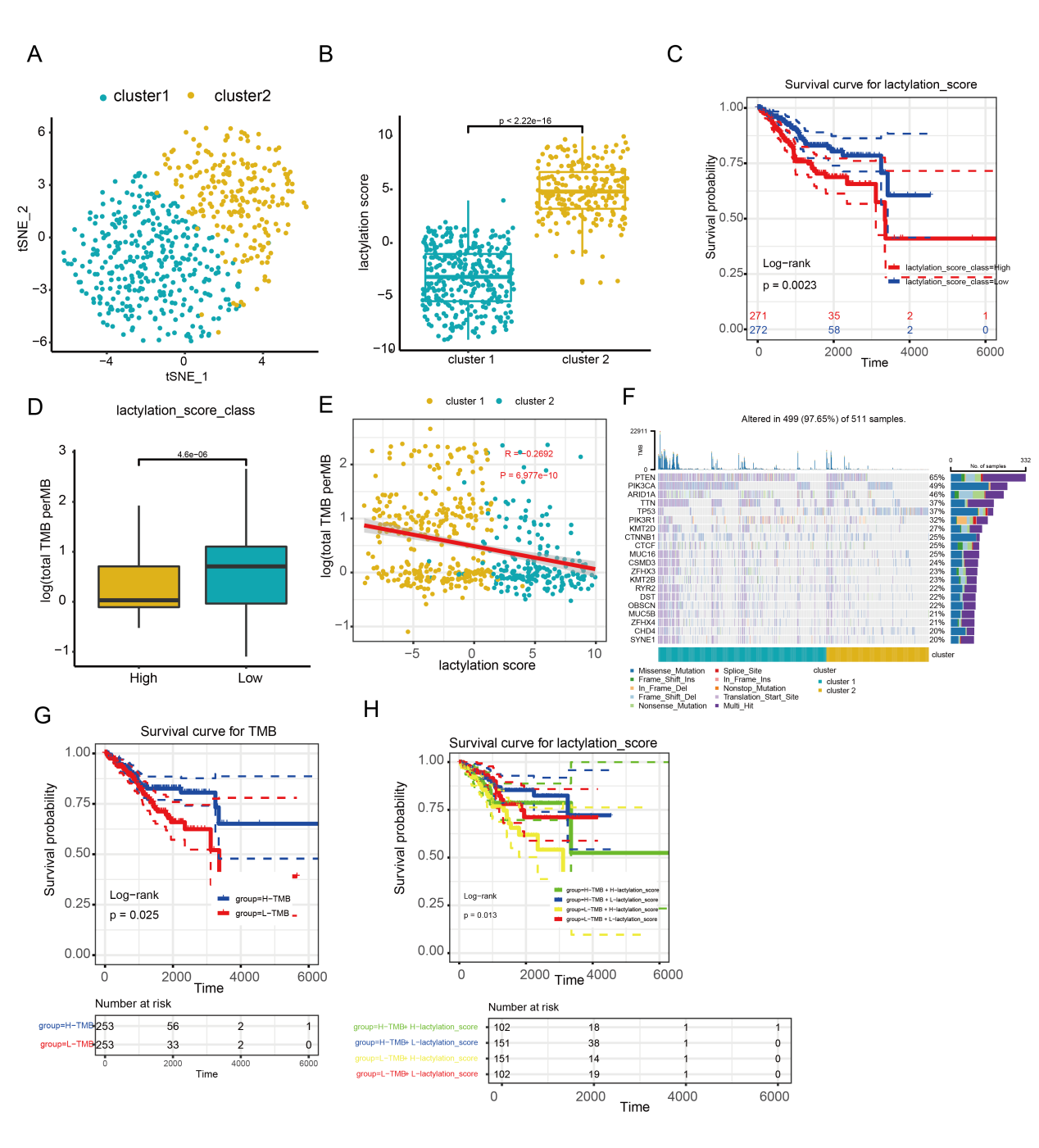


**Figure S3 Lactylation score model**

(A) tSNE dimensionality reduction for lactylated genes. (B) Lactylation scores of the two clusters. (C) Kaplan–Meier survival curves of the high- and low-lactylation score groups. (D) TMB scores of the high- and low-lactylation score groups. (E) Spearman’s correlation analysis of the lactylation and TMB scores. (F) Waterfall plot of the mutation frequencies. (G) Kaplan–Meier survival curves of the high and low TMB groups. (H) Joint survival analysis was performed for the high or low TMB and high- or low-lactylation score groups.

**
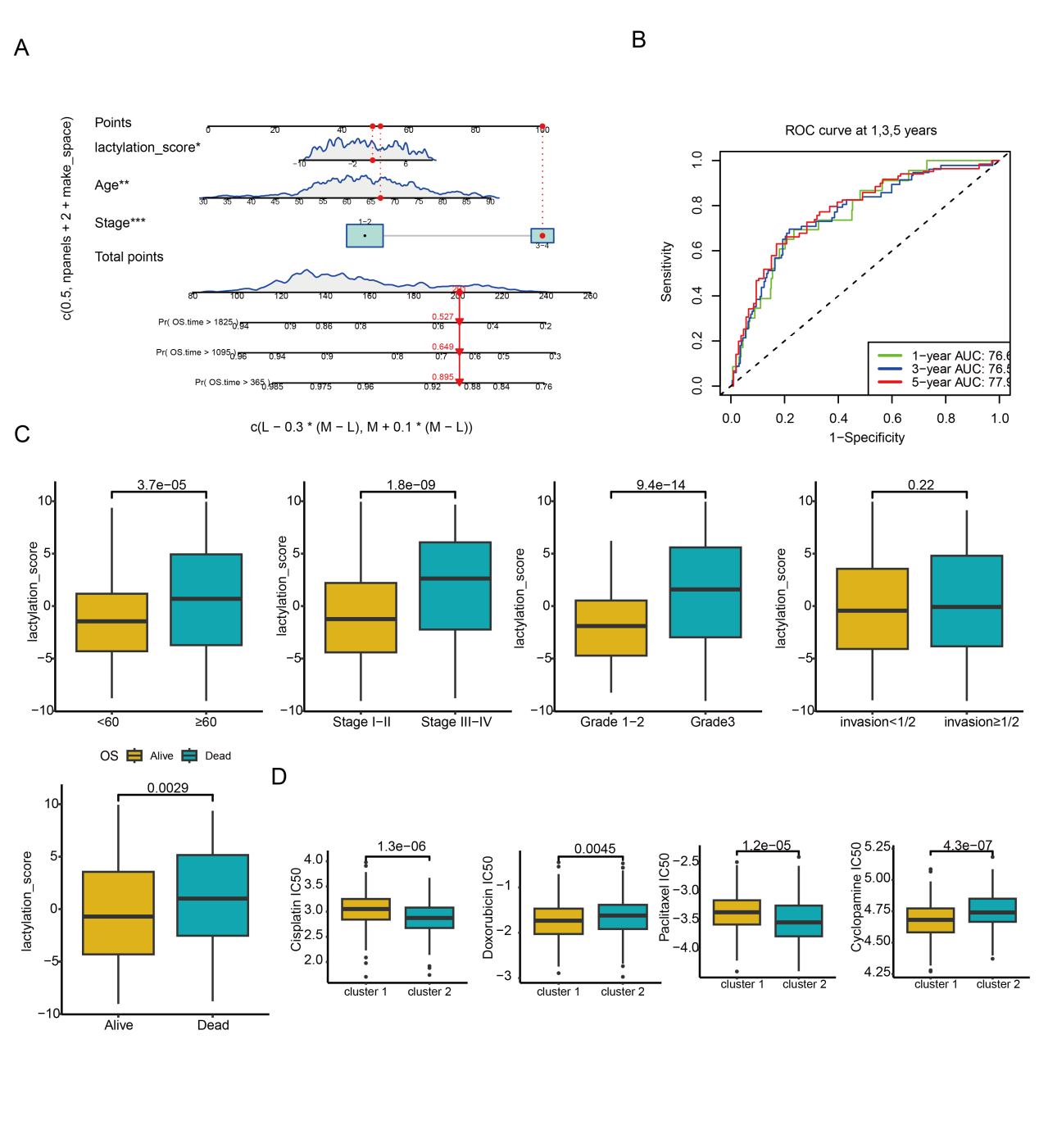
**

**Figure S4 Lactation score and pathological feature model.**

(A) Nomogram of the lactylation score and clinical information. (B) AUC of the lactylation score model. (C) Age, stage, grade, invasion, and survival of patients in the high and low lactylation score groups. (D) Differences in the sensitivity of subtypes to chemotherapy, namely cisplatin, doxorubicin, paclitaxel, and cyclopamine, are shown for the two clusters.


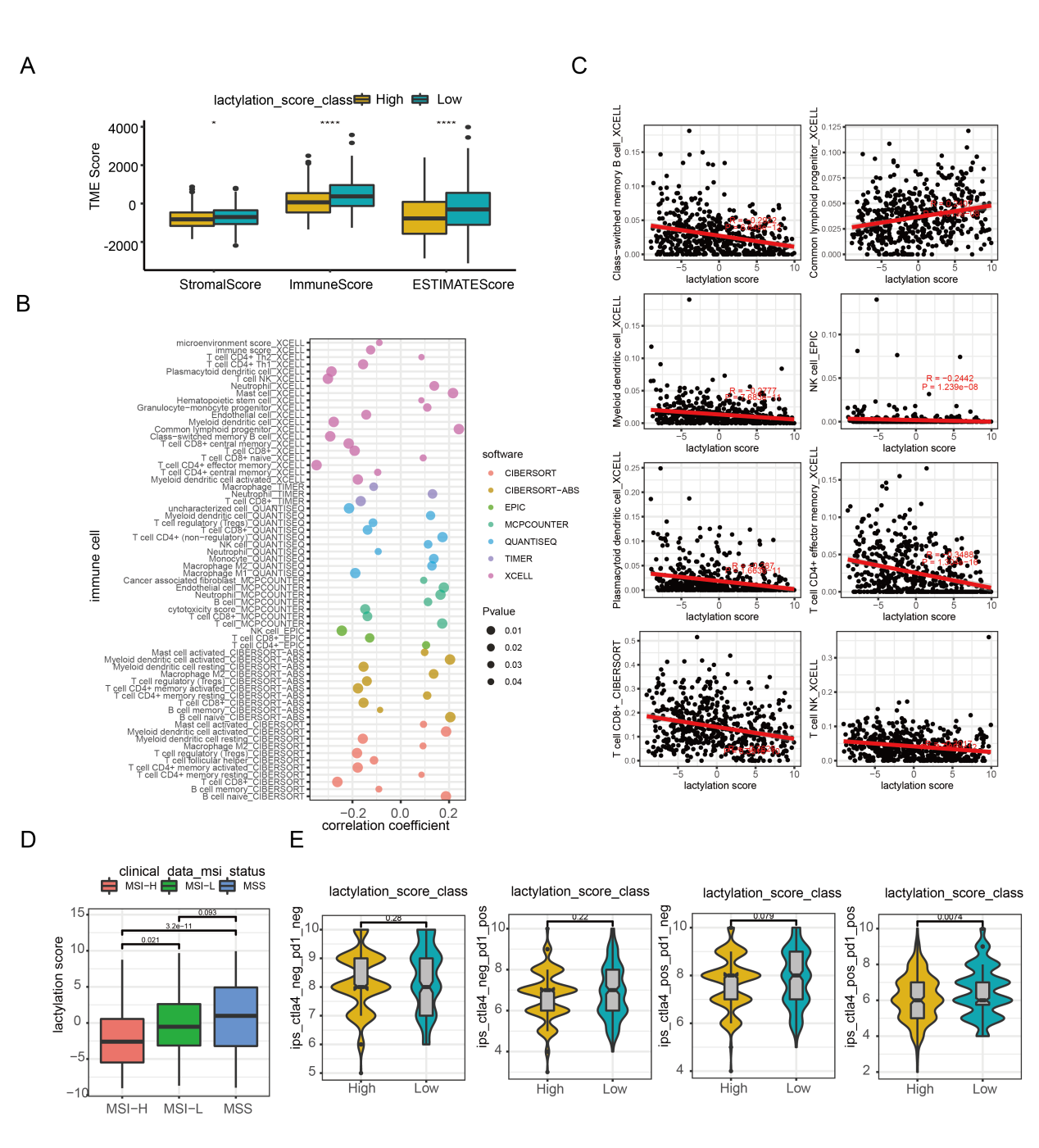


**Figure S5 Immune cell infiltration and function enrichment analysis**

(A) Correlation between lactylation score and the tumour microenvironment of

EC was assessed using the ESTIMATE algorithm. (B-C) Correlation between lactylation score and immune cell infiltration using various immunocyte analysis methods. (D) Microsatellite instability in the high- and low-lactylation score groups. (E) Sensitivity analysis of the high- and low-lactylation score groups to immunotherapy.

**Abbreviations**

EC, endometrial cancer

UCEC, uterine corpus endometrial carcinoma

OS, overall survival

ssGSEA, single sample gene set enrichment analysis

TCGA, The Cancer Genome Atlas

GSEA, Gene Set Enrichment Analysis

GSVA, Gene set variation analysis

TMB, tumour mutation burden

PFKM, phosphofructokinase, muscle

MECP2, methyl CpG binding protein 2

qRT-PCR, quantitative reverse transcription polymerase chain reaction

LC/MS, liquid chromatography/mass spectrometry

TILs, tumour infiltrating lymphocytes

MSI, microsatellite instability

MSI-H, MicroSatellite Instability-High

MSS, MicroSatellite stability

GDSC, Genomics of Drug Sensitivity in Cancer

IC_50_, half-maximum inhibitory concentration

ES, EnrichmentnScore

TME Score, Tumor microenviroment Score

Supplementary Table S2

The expression of PFKM in normal endometrial tissue and [endometrial carcinoma](javascript:void(0)) tissue

| Group | n | Positive Expression of PFKM n(%) | | *P* |
| --- | --- | --- | --- | --- |
| Normal endometrial tissue | 8/20 | 40% | < 0.05 | |
| [endometrial carcinoma](javascript:void(0)) tissue | 21/30 | 70% |  | |

**Acknowledgements**

The authors thank their laboratory staff and collaborators for supporting this research.
