# Supplementary material for: Role of PFKM lactylation in glycolysis regulation in endometrial cancer cells
Source: Genes Dis. 2024 Aug 30;12(3):101400. doi: 10.1016/j.gendis.2024.101400 (PMC11786832; doi:10.1016/j.gendis.2024.101400)
Supplement: Multimedia component 2 [file mmc2.docx]

Additional file 1: Table S1

Table S1: Primer sequences for qRT-PCR and Primary antibodies used for the detection of protein expression.

| Name | Sequence |
| --- | --- |
| PFKM | F: TCACAGATGAGGAGGCTACGAAGTC  R: GGTCATATCGGTGCCACAGAAGTC |
| MECP2 | F: TCGGTGAGAAGAGCGGGAAAGG  R: TCTGAGTGGTGGTGATGGTGGTG |
| MDH1  GPI | F: AGAAGGGAAGGCATGGAGAGAAAAG  R: AAGCAGTCAGGCAGTTGGTATTGG  F: TGCCAACAAGGACCGCTTCAAC  R: GTCCACCAGCATCCGCATCAC |
| actin  PFKM  lactylation | F: CCTGGCACCCAGCACAAT R: GGGCCGGACTCGTCATAC  Manufacturer: Proteintech  Dilution ratio: Immunohistochemistry: 1:50  Manufacturer: Jingjie PTM BioLabs, Hangzhou, China  Dilution ratio: western blotting, 1:500 |
