# Supplementary material for: Role of PFKM lactylation in glycolysis regulation in endometrial cancer cells
Source: Genes Dis. 2024 Aug 30;12(3):101400. doi: 10.1016/j.gendis.2024.101400 (PMC11786832; doi:10.1016/j.gendis.2024.101400)
Supplement: Multimedia component 3 [file mmc3.docx]

Supplementary Table S2

The expression of PFKM in normal endometrial tissue and [endometrial carcinoma](javascript:void(0)) tissue

| Group | n | Positive Expression of PFKM n(%) | | *P* |
| --- | --- | --- | --- | --- |
| Normal endometrial tissue | 8/20 | 40% | < 0.05 | |
| [endometrial carcinoma](javascript:void(0)) tissue | 21/30 | 70% |  | |
